# Classic IL-6R signalling is dispensable for intestinal epithelial proliferation and repair

**DOI:** 10.1038/oncsis.2016.71

**Published:** 2016-11-21

**Authors:** K Aden, A Breuer, A Rehman, H Geese, F Tran, J Sommer, G H Waetzig, T M Reinheimer, S Schreiber, S Rose-John, J Scheller, P Rosenstiel

**Affiliations:** 1 Institute of Clinical Molecular Biology, University Hospital Schleswig-Holstein, Campus Kiel, Kiel, Germany; 2 First Medical Department, University Hospital Schleswig-Holstein, Campus Kiel, Kiel, Germany; 3 Institute of Biochemistry and Molecular Biology II, Medical Faculty, Heinrich-Heine-University, Düsseldorf, Germany; 4 CONARIS Research Institute AG, Kiel, Germany; 5 Ferring Pharmaceuticals A/S, Copenhagen, Denmark; 6 Institute of Biochemistry, Kiel University, Kiel, Germany

## Abstract

Inflammatory bowel disease is characterized by disturbed cytokine signalling in the mucosa. Inhibition of the proinflammatory interleukin (IL)-6 pathway is a promising new therapeutic strategy, but safety concerns arise as IL-6 signalling also contributes to epithelial repair of the intestinal mucosa. To which extent IL-6 classic or trans-signalling contributes to intestinal repair remains elusive. We tested the influence of IL-6 classic signalling on intestinal repair and proliferation. Whereas IL-6 induced STAT3 phosphorylation in the colonic cancer cell lines, primary non-malignant intestinal organoids did not respond to IL-6 classic signalling. Mice deficient in intestinal IL-6R (IL-6R^ΔIEC^ mice) did not display increased susceptibility to acute dextran sulfate sodium (DSS)-induced colitis. In the azoxymethane DSS model IL-6R^ΔIEC^ mice were not protected from inflammation-induced carcinogenesis but showed comparable tumor load to wild-type mice. These data indicate that classic signalling is not the major pathway to transduce IL-6 stimuli into the intestinal epithelium.

## Introduction

Proficient epithelial regeneration is a prerequisite to maintain intestinal homeostasis.^1^ Within the intestinal epithelium, regeneration is elicited by pro-regenerative signals, which either derive from invading immune cells or are secreted in a paracrine and autocrine manner from intestinal epithelial cells (IECs).^2^ Many cytokines that elicit proliferative signals in the intestinal epithelium (for example, interleukin (IL)-17, IL-22, IL-11, IL-10 or IL-36) employ epithelial Janus kinase/signal transducer and activator of transcription (JAK/STAT) signalling, mostly by engaging STAT3 as the major signal transducer.^3, 4, 5^ Mice lacking epithelial STAT3 or JAK3 signalling display impaired epithelial regeneration^6, 7^ and increased T cell-driven intestinal inflammation.^8^ On the other hand, overactivation of the JAK/STAT pathway renders mice more susceptible to colon cancer formation, which highlights the dual role of JAK/STAT in tightly controlling intestinal regeneration.^9^

IL-6 is a key cytokine in intestinal inflammation with pleiotropic functions both as a pro-inflammatory and as a regeneration-promoting factor.^10, 11^ IL-6 also activates JAK/STAT signalling, mainly by STAT3. Upon binding of IL-6 to the membrane-bound IL-6 receptor (IL-6R), a dimer of the IL-6 family signal transducer glycoprotein gp130 is recruited, which leads to subsequent intracellular autophosphorylation of the gp130-associated tyrosine kinase Jak1 and finally to phosphorylation and activation of STAT3.^12^

While all body cells express the signal-transducing beta-receptor gp130, only few cell types (mainly hepatocytes and some leukocytes) express the non-signalling, specific IL-6R.^13^ Activation of gp130 via the membrane-bound IL-6R is called 'classic signalling'. Soluble IL-6R (sIL-6R) is mainly produced by protease shedding of the IL-6R ectodomain and, to a minor extent, by alternative splicing, and IL-6/sIL-6R complexes can activate also cells only expressing gp130, that is, practically all cells of the body. This process is called 'trans-signalling' and is thought to be a major sensitizing pathway for chronic inflammation.^14^ IL-6 trans-signalling contributes to intestinal proliferation, and overshooting IL-6 trans-signalling leads to murine colon cancer formation.^15^

IL-6 as a therapeutic target is currently under investigation in various chronic immune-mediated diseases, including rheumatic arthritis and inflammatory bowel disease (IBD). The interest in using IL-6 blockade as a therapeutic strategy in IBD is driven by the finding that the pathophysiology of IBD is strongly influenced by host genetics and many enriched genes are involved in pathways that influence host−environmental interactions and intestinal immune homeostasis.^16, 17, 18^ A small phase 2 trial showed favorable results, but no mucosal healing.^19^ Whereas IL-6 blockade in rheumatic arthritis has been shown to be safe and efficacious,^20^ concerns about the safety of IL-6 blockade in the context of intestinal inflammation were raised by various findings: (i) the incidence of intestinal perforations in rheumatic arthritis patients is increased under therapy with tocilizumab (Tcz), a human anti-IL-6R antibody, when compared to therapy with conventional disease-modifying anti-rheumatic drugs;^21^ (ii) IL-6R salvage therapy induces exacerbation in ulcerative colitis with increased ulcer formation;^22^ (iii) a clinical phase 2 study testing the anti-IL-6 agent BMS-945429 in Crohn's disease had to be prematurely terminated due to two cases of intestinal perforations (Clinicaltrials.gov Identifier: IM133-055). These observations raised questions about the role of IL-6 signalling in mediating intestinal regeneration. Whereas the role of common (classic and trans) IL-6 signalling in the context of intestinal regeneration has been studied,^11^ the contribution of specific IL-6 classic signalling in intestinal epithelial regeneration remains disputable. Although IL-6 classic signalling has been described for intestinal epithelial tumor cell lines,^23, 24^ the influence of IL-6 classic signalling on intestinal proliferation *in vivo* is rather circumstantial.^25, 26^ More precisely, very few studies have shown a biological effect of IL-6 classic signalling on epithelial proliferation.^26^

By employing *in vitro* methods of intestinal regeneration and *in vivo* mouse models of experimental colitis using a conditional deletion of the IL-6R in the intestinal epithelium (IL-6R^ΔIEC^), we therefore investigated the influence of epithelial IL-6R on intestinal proliferation and repair.

## Results and discussion

### IECs receive IL-6 signals via classic and trans-signalling

**Figure 1:**
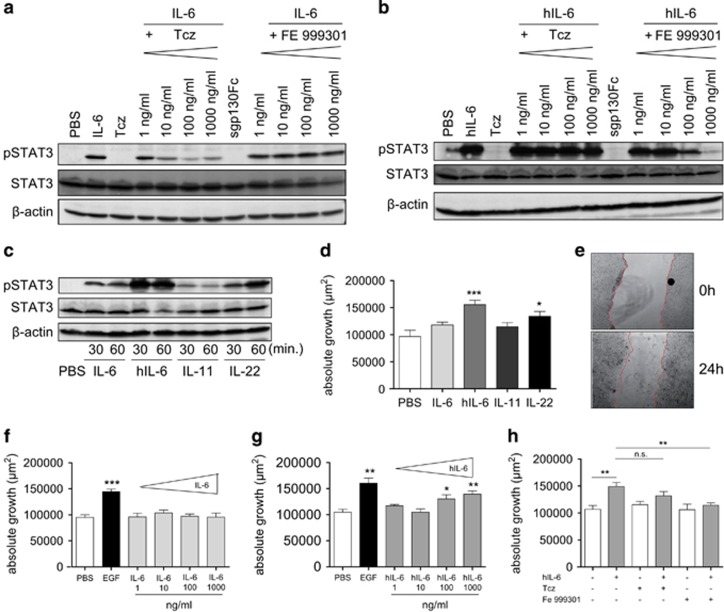
Functional classic IL-6 signalling in IECs does not lead to increased cellular proliferation. (**a**) Tcz inhibits STAT3 phosphorylation (pSTAT3) after induction of classic signalling in a dose-dependent manner. Western blot analysis of lysates from HT-29 colon carcinoma cells, pre-treated with Tcz (1–1000 ng/ml) or sgp130Fc (1–1000 ng/ml) for 6 h and stimulated with human IL-6 (100 ng/ml) for 30 min. (**b**) sgp130Fc dose-dependently blocks trans-signalling: western blot analysis of lysates from HT-29 colon carcinoma cells, pre-treated with Tcz or sgp130Fc (1–1000 ng/ml) for 6 h and stimulated with hyper-IL-6 (hIL-6) (10 ng/ml) for 30 min. (**c**) HT-29 colon carcinoma cells were stimulated with IL-6 (100 ng/ml), hIL-6 (100 ng/ml), IL-11 (100 ng/ml) or IL-22 (100 ng/ml) for 30 or 60 min, and protein lysates were probed for (p)STAT3, STAT3 or β-actin. (**d**) IL-6 trans-signalling and IL-22, but not IL-6 classic signalling or IL-11 induce epithelial regeneration: confluent HT-29 cells (*n*=8 wells/stimulation) were scratched with a sterile 200 μl pipette and stimulated with IL-6 (100 ng/ml), hIL-6 (100 ng/ml), IL-11 (100 ng/ml) or IL-22 (100 ng/ml) for 24 h. Absolute and relative growth was assessed 24 h after scratching. (**e**) Representative photomicrographs were taken at 0 and 24 h after scratch induction in PBS-treated HT-29 cells. (**f**) IL-6 classic signalling does not alter intestinal epithelial proliferation and migration: absolute growth of HT-29 colon carcinoma cells after scratching with a sterile pipette and treatment with human EGF (10 ng/ml) or IL-6 (1–1000 ng/ml) for 24 h (*n*=8 wells per stimulation). (**g**) IL-6 trans-signalling increases intestinal epithelial proliferation and migration: HT-29 colon carcinoma cells were scratched and stimulated with human EGF (10 ng/ml) or hIL-6 (1–1000 ng/ml), and absolute growth was assessed after 24 h (**f**). (**h**) sgp130Fc, but not Tcz, inhibits hIL-6-induced epithelial proliferation and migration: HT-29 colon carcinoma cells were pre-incubated with Tcz (1000 ng/ml) or sgp130Fc (1000 ng/ml) for 4 h, scratched and stimulated with hIL-6 (100 ng/ml), and absolute growth was assessed after 24 h (*n*=8 wells per stimulation). Note the slight variations of absolute growth between **g**, **h**, which results from biological replicates of the experiment. Data are representative of *n*=2 individual experiments. Significance was determined using the two-tailed Student's *t*-test, and data are expressed as mean±s.d. *P<0.05; **P<0.01; ***P<0.001.

To test whether IECs receive IL-6 signals via classic and/or trans-signalling, human HT-29 colonic carcinoma cells were stimulated with different concentrations of IL-6 to activate the classic signalling pathway. Alternatively, trans-signalling was activated in these cells by using hyper-IL-6 (hIL-6), a fusion protein consisting of human IL-6 linked by a flexible peptide chain to human sIL-6R.^27, 28^ Cells were stimulated for 30 min, with or without pre-incubation with Tcz, a neutralizing human anti-IL6R antibody, or optimized sgp130Fc, a fusion protein of the extracellular domain of gp130 dimerized by the Fc domain of human IgG1 that selectively blocks trans-signalling.^29, 30^ IL-6 stimulation induced phosphorylation of STAT3, which was inhibited by pre-incubation with Tcz in a dose-dependent manner. As expected, pre-incubation with sgp130Fc did not interfere with IL-6-induced STAT3 phosphorylation (Figure 1a). Conversely, hIL-6-induced STAT3 phosphorylation was inhibited in a dose-dependent manner by pre-treatment with sgp130Fc, whereas Tcz had no inhibitory effect on hIL-6-induced STAT3 phosphorylation (Figure 1b), which is in line with previous observations and is due to the fact that Tcz, which is designed to block binding of IL-6 to (s)IL-6R, cannot significantly interfere with any formation of the already covalently formed IL-6/sIL-6R complex.^31^ To further investigate the role of classic and trans-signalling in epithelial wound healing *in vitro*, we assessed the effect of IL-6 and hIL-6 in an epithelial scratch assay in HT29 cells^32^ using IL-6 and hIL-6 concentrations, which were able to induce STAT3 in a robust manner. We also used IL-11 and IL-22 for comparison, as those cytokines have also been described to induce epithelial proliferation.^33, 34^ Interestingly, whereas all cytokines were able to induce STAT3 phosphorylation with different efficacies and kinetics (Figure 1c), only hIL-6 and IL-22 were able to induce a moderate, but statistically significant increase of intestinal wound healing (Figure 1d). Wound healing was assessed as the reduction of the area between the wound edges, as shown in representative micrographs of wound areas before (0 h) and 24 h after wound induction (Figure 1e). IL-11 or IL-6 did not show any effect on intestinal epithelial regeneration.

We further performed a detailed titration of IL-6 and hIL-6 in the scratch assay to determine whether higher doses of the cytokines would lead to increased proliferation and migration. However, the results again indicated that IL-6 classic signalling did not induce intestinal epithelial proliferation and migration (Figure 1f). In contrast, hIL-6 was able to induce wound healing, albeit at very high concentrations (100 or even 1000 ng/ml, corresponding to 1.67 or 16.7 nM, respectively) (Figure 1g). For comparison, a maximum cell proliferation of the very hIL-6-responsive cell line Ba/F3-gp130,^35, 36^ is achieved with 10 ng/ml hIL-6, similar to the 10 ng/ml EGF giving a maximum reference signal in the scratch assay used in the present study (Figures 1f and g). Lastly, we could show that only sgp130Fc, but not Tcz, could inhibit the hIL-6 induced wound healing (Figure 1h).

### Intestinal organoids respond to IL-6 trans-signalling, but not to classic signalling

The results described above prompted us to test the impact of the presence of the epithelial membrane-bound IL-6R on IL-6-dependent intestinal regeneration in a clean genetic model of epithelial IL-6R deletion. For this purpose, we crossed *IL-6R*^*flox*^ mice^37^ with C57Bl/6^VillinCre^ mice to generate conditional knockouts in the intestinal epithelium, which were termed *IL-6R*^*ΔIEC*^ (for ‘deleted in intestinal epithelial cells'). *IL-6R*^*fl/fl*^ (*IL-6R*^*fl*^) littermates were used as controls.

**Figure 2:**
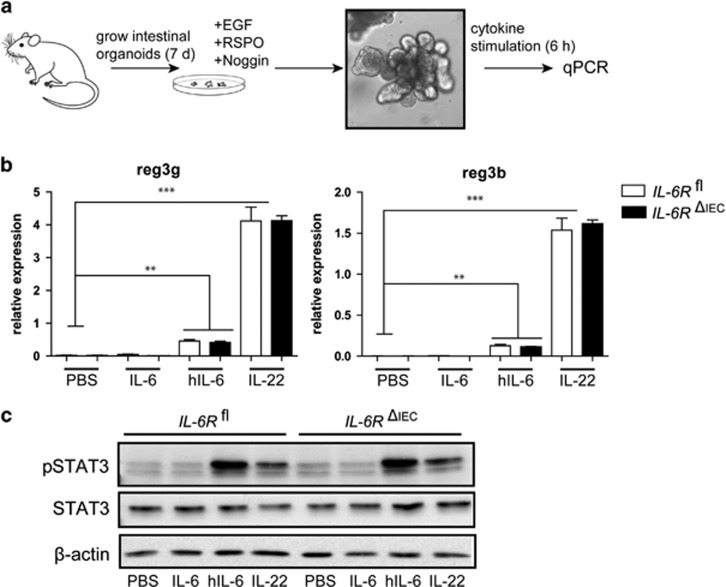
IL-6 trans-signalling, but not classic signalling, induces STAT3 target gene expression in intestinal organoids. (**a**) Mouse intestinal organoids from *IL-6R*^*fl*^and *IL-6R*^*ΔIEC*^ mice were cultivated as described previously.^21^ The medium was changed every other day and organoids were stimulated after 7 days of cultivation with hIL-6 (100 ng/ml), IL-6 (100 ng/ml) or IL-22 (100 ng/ml). (**b**) Expression of Reg3b and Reg3 was assessed by qPCR, and data are expressed as the expression relative to the housekeeping gene β-actin. (**c**) Western blot of isolated IECs, stimulated for 30 min with hIL-6 (100 ng/ml), IL-6 (100 ng/ml) or IL-22 (100 ng/ml) and probed for pSTAT3, STAT3 and β-actin. Data are representative of *n*=2 individual experiments. Significance was determined using the two-tailed Student's *t*-test, and data are expressed as mean ± s.d. **P<0.01; ***P<0.001.

IL-6 has been shown to act as a canonical STAT3 inducer in multiple colon cancer epithelial cell lines.^23^ As epithelial regenerative responses occur independently from malignant transformations, we wanted to assess the role of IL-6 classic signalling in a epithelial non-tumor-derived model. For this purpose we cultured intestinal organoids from *IL-6R*^*fl*^ and *IL-6R*^*ΔIEC*^ mice as described previously^38^ (Figure 2a). Intestinal organoids were stimulated with IL-6, hIL-6 or IL-22, and the expression of the STAT3 target genes *Reg3b* and *Reg3g* was analyzed. Whereas IL-22 and hIL-6 induced a strong expression of STAT3 target genes, IL-6 did not induce any STAT3-driven gene expression (Figure 2b). Interestingly, no difference was seen between intestinal organoids from *IL-6*R^*fl*^ and *IL-6R*^*ΔIEC*^ mice, indicating that, if at all, the epithelial IL-6R does not interact with IL-6 trans-signalling. To further delineate whether impaired expression of Reg3b and Reg3g is indeed a surrogate of impaired phosphorylation of STAT3, IECs were stimulated with indicated cytokines for 30 min and probed for pSTAT3. Indeed, only hIL-6 and IL-22 induced phosphorylation of STAT3, whereas IL-6-stimulated cells showed no response (Figure 2c). This finding demonstrates that in non-malignant epithelial cells the epithelial IL-6R does not transduce STAT3-driven signals into the epithelium via classic IL-6R signalling.

### *Il6-R*^*ΔIEC*^ mice do not display increased susceptibility to dextran sulfate sodium (DSS)-induced colitis

**Figure 3:**
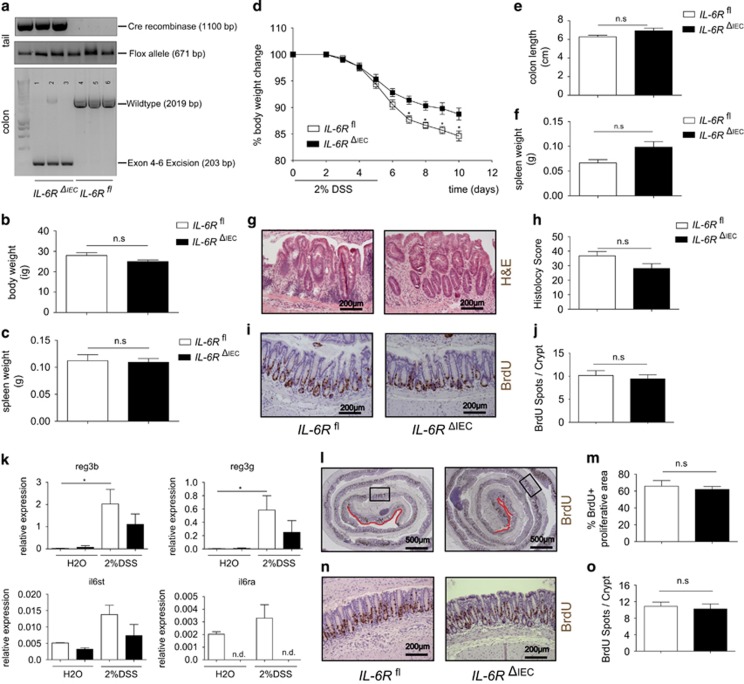
*IL-6R*^*ΔIEC*^ are not susceptible to DSS colitis and display regular epithelial regeneration. (**a**) Genotyping of *IL-6R*^*fl*^ and *IL-6R*^*ΔIEC*^, showing expression of Cre recombinase and the mutant IL-6R allele in tail PCR. IEC-specific deletion of IL-6R was verified by isolating colon DNA and using primer pairs spanning the exon 4–6 region of the IL-6R gene (*il6ra*). PCR products show an amplicon of 2016 bp in *IL-6R*^*fl*^ and a truncated amplicon of 203 bp in *IL-6R*^*ΔIEC*^plus a faint 2016 bp signal, indicating DNA from non-epithelial origin. (**b**, **c**) Body weight and spleen weight of sex-matched untreated *IL-6R*^*f*^(*n*=4) mice and *IL-6R*^*ΔIEC*^ (*n*=4) at the age of 8–12 weeks. (**d**) Sex-matched littermate *IL-6R*^*f*l^ (*n*=8) and *IL-6R*^*ΔIEC*^ (*n*=18) mice aged 8–12 weeks were subjected to experimental colitis induced by adding 2% of DSS to the drinking water. Body weight changes relative to the starting weight on day 0 showed that *IL-6R*^*ΔIEC*^ mice lost less weight than their *IL-6R*^*fl*^ littermates. (**e**, **f**) Postmortem analyses showed no significant difference between *IL-6R*^*ΔIEC*^ and *IL-6R*^*fl*^ in colonic shortening (**e**) or relative spleen weight (**f**). (**g**, **h**) H&E staining of colon Swiss rolls (**g**) and corresponding histological disease scores^42^ (**h**) showed no significant differences in disease severity between *IL-6R*^*ΔIEC*^ and *IL-6R*^*fl*^mice. (**i**, **j**) Representative pictures of BrdU staining of *IL-6R*^*ΔIEC*^ and *IL-6R*^*fl*^ are shown in (**i**). 10 mg/kg bodyweight of BrdU was injected i.p. 1.5 h before sacrifice. A minimum of 15 crypts/intestine was counted and genotype pooled data from *IL-6R*^*f*l^ (*n*=6) and *IL-6R*^*ΔIEC*^ (*n*=12) are depicted for statistical evaluation. No significant difference was seen in the number of BrdU+ cells/crypt between *IL-6R*^*ΔIEC*^ and *IL-6R*^*fl*^ mice (**j**). (**k**) IEC-specific expression of STAT3 target genes. Mice (*n*>3 per genotype) were challenged with 2% DSS or left untreated (water, H2O) for 3 days, and colonic IECs were isolated. Gene expression was assessed using qPCR. DSS induction led to significant upregulation of STAT3 target genes in *IL-6R*^*fl*^ animals, which was reduced or abrogated in *IL-6R*^*ΔIEC*^ mice. (**l**, **m**) Representative pictures of BrdU staining of *IL-6R*^*ΔIEC*^ and *IL-6R*^*fl*^ are shown in (**i**). 10 mg/kg bodyweight of BrdU was injected i.p. 1.5 h before sacrifice. The percentage of intact BrdU^+^ mucosa was expressed as 1−(length of BrdU-negative mucosa in μm/total mucosal length in μm)*100. (**n**, **o**) A minimum of 15 crypts/intestine were evaluated for BrdU-positive stained cells. Genotype pooled data from *IL-6R*^*f*l^ (*n*=4) and *IL-6R*^*ΔIEC*^ (*n*=4) are depicted for statistical evaluation. Significance was determined using the two-tailed Student's *t*-test, and data are expressed as mean±s.d. *P<0.05.

Having shown that classic IL-6 signalling is not active in non-malignant IECs *in vitro*, we wanted to test the role of the epithelial IL-6R *in vivo*. Deletion efficiency and specificity were tested in genotyping PCR from tail and colon IEC DNA (Figure 3a). Macromorphological analysis of the small and large intestine of *IL-6*R^*ΔIEC*^ mice did not reveal any spontaneous abnormalities when compared to wild-type littermates (data not shown), and baseline body weight (Figure 3b) and spleen weight (Figure 3c) were comparable between age- and sex-matched littermates. To determine the impact of IL-6R signalling on intestinal proliferation and repair under exogenous stress, we exposed *IL-6R*^*fl*^ and *IL-6R*^*ΔIEC*^ mice to DSS, a chemical irritant that disrupts the intestinal epithelial barrier and results in induction of colitis.^39^ Mice of both genotypes started to lose weight, but *IL-6R*^*ΔIEC*^ mice seemed to cope better with DSS-induced colitis than their wild-type littermates (Figure 3d) as they significantly lost less weight than *IL-6R*^*fl*^ mice. Remarkably, *IL-6R*^*ΔIEC*^ lost significantly less weight than the *IL-6R*^*fl*^ littermates, but showed no significant differences in other parameters of inflammation. Next, we aimed to rule out environmental factors that could influence the overall outcome of the DSS colitis. We performed 16srRNA sequencing of the luminal microflora after DSS colitis. *IL-6R*^*fl*^ and *IL-6R*^*ΔIEC*^ showed no difference in the overall composition of the colonic microflora and the relative abundance of major phylotypes (data not shown). In post-mortem analyses, no significant difference was seen in colon length (Figure 3e) or spleen weight (Figure 3f), although a non-significant trend points towards a diminished inflammatory response in *IL-6*R^*ΔIEC*^ mice.This was in line with histological evaluation of the diseased colon, which showed a non-significant trend towards less histological inflammation in *IL-6*R^*ΔIEC*^ in the overall histological score (Figures 3g and h) or in any subscore (mononuclear infiltration, crypt hyperplasia, epithelial erosion, polymorphonuclear infiltrates, transmural inflammation; details not shown). In order to investigate the impact of IL-6R signalling on intestinal regeneration, we also evaluated the overall bromodeoxyuridine-positive (BrdU^+^) areas of the entire colon. Interestingly, *IL-6R*^*ΔIEC*^ mice showed significantly increased mucosal areas with BrdU incorporation, thereby indicating more proficient epithelial regeneration than *IL-6R*^*fl*^ littermates. (Figures 3i and j). It must be noted that the acute DSS model is driven by an acute toxic destruction of the intestinal epithelium, in which partial IL-6-dependent regenerative responses can be completely overridden by the overt inflammation. In order to describe presumable partial regenerative effects of the epithelial IL-6R on the intestinal epithelium, a DSS colitis was induced only for 3 days. Colonic IECs were isolated and tested for STAT-dependent gene expression. DSS induction induced an upregulation of the STAT3 target genes *Reg3g* and *Reg3b* in *IL-6R*^*ΔIEC*^ and *IL-6R*^*fl*^, indicating that epithelial IL-6R is not primarily involved in epithelial STAT3 signalling. As expected, Il6ra expression was absent in epithelial cells of *IL-6R*^*ΔIEC*^ compared to *IL-6R*^*fl*^, whereas gp130(IL6st) expression levels in IECs were comparable (Figure 3k). BrdU staining revealed again comparable numbers of proliferative cells in the colon mucosa of *IL-6R*^*ΔIEC*^ and *IL-6R*^*fl*^ (Figures 3l–o). Although we cannot entirely rule out an incremental beneficial effect of IL-6 signalling on the intestinal epithelium, our data support the hypothesis that epithelial IL-6R does not orchestrate the regenerative response of the intestinal mucosa to inflammatory conditions.

### *IL-6R*^*ΔIEC*^ mice are not protected from azoxymethane (AOM)-DSS-induced carcinogenesis

**Figure 4:**
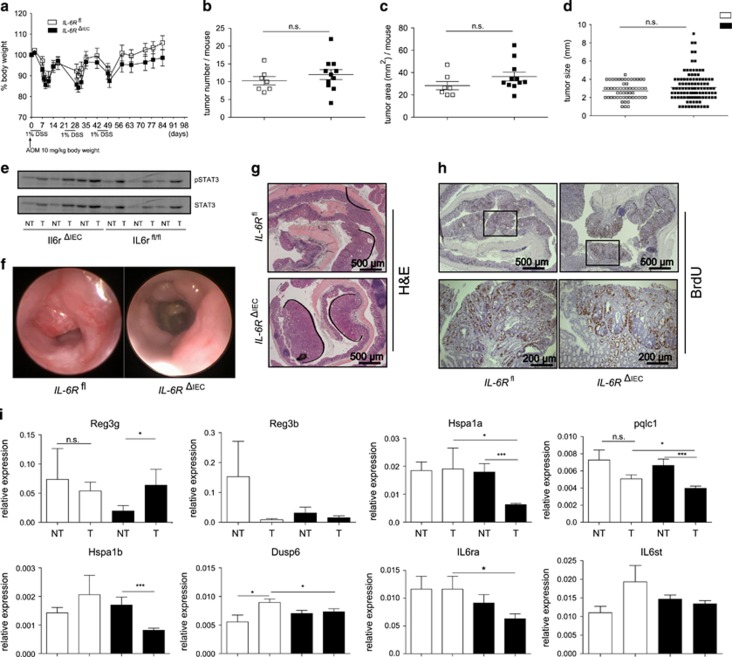
*IL-6R*^*ΔIEC*^ mice are not protected from colitis-associated cancer. (**a**) Sex-matched littermate *IL-6R*^*fl*^ (*n*=9) and *IL-6R*^*ΔIEC*^ (*n*=16) mice aged 8–12 weeks were subjected to an AOM-DSS colitis model. A single dose of 10 mg/kg of AOM was injected at day 0, followed by three cycles of 1% DSS added to the drinking water. Body weight changes relative to the starting weight on day 0 were not different between *IL-6R*^*ΔIEC*^ and *IL-6R*^*fl*^ mice. (**b**) Absolute number of tumors/mouse. (**c**) Average tumor area/mouse. (**d**) Overall distribution of tumor size between genotypes. (**e**) Protein lysates from colonic tumor and adjacent non-tumor tissue were probed on western blot against pSTAT3 and STAT3. (**f**) Representative pictures of mouse colonoscopy at the day of sacrifice showing no difference in endoscopic tumor growth between *IL-6R*^*ΔIEC*^ and *IL-6R*^*fl*^mice. (**g**, **h**) Representative histological pictures of colon Swiss rolls of H&E or BrdU staining revealed no difference in histological tumor growth or tumor proliferation between *IL-6R*^*ΔIEC*^ and *IL-6R*^*fl*^mice. (**i**) Gene expression patterns in tumor vs non-tumor colon tissues in *IL-6R*^*ΔIEC*^ and *IL-6R*^*fl*^ mice showed significant changes. Significance was determined using the two-tailed Student's *t*-test, and data are expressed as mean±s.d. *P<0.05; ***P<0.001.

It has been recently postulated that classic IL-6R signalling contributes to intestinal carcinogenesis in a model of inflammasome-triggered carcinogenesis.^40^ We therefore tested the hypothesis that deletion of the IL-6R in IECs protects from inflammation-induced carcinogenesis using the AOM-DSS model. Mice received a single dose of 10 mg/kg AOM on day 0, and chronic DSS colitis was induced using three cycles of 1% DSS (Figure 4a). During the colitis cycles four *IL-6*R^*ΔIEC*^ and two *IL-6R*^*fl*^ mice died prematurely (data not shown). Postmortem analysis revealed no difference in total numbers of tumors/mouse (Figure 3b), total tumor area/mouse (Figure 3c) or in tumor size (Figure 3d) between *IL-6R*^*ΔIEC*^ and *IL-6R*^*fl/fl*^ mice. Western blot of mucosa from tumors or adjacent non-tumor mucosa showed increased tumor-dependent STAT3 phosphorylation levels. In line, no genotype-specific differences were seen, which indicates that classical IL-6R signalling does not contribute to malignant STAT3 activation in colon carcinogenesis (Figure 3e). Endoscopic assessment of tumors showed, again, no difference between *IL-6R*^*ΔIEC*^ and *IL-6R*^*fl*^ mice (Figure 3f). Histopathological assessment showed no difference in histological H&E staining (Figure 3g) or in BrdU staining in colonic tumors (Figure 3h). As these data suggested that epithelial-specific deletion of the IL-6R does not inhibit carcinogenesis, we investigated the impact of impaired classic IL-6 signalling in the context of tumor or non-tumor lesions. IL-6 has been described to induce a specific set of genes in the context of intestinal carcinogenesis, which is distinct from the STAT3-signalling cytokines like IL-11.^34^ Indeed, a set of genes were exclusively downregulated in tumors from *IL-6*R^*ΔIEC*^ but not *IL-6*R^*fl*^ mice, suggesting that IL-6 signalling is biologically active in the context of intestinal carcinogenesis but does not affect the overall tumor development (Figure 3i). Interestingly, gene expression of IL-6R itself (il6ra) was still detectable in tumor and non-tumor mucosal specimens from *IL-6R*^*ΔIEC*^ mice, which was most probably due to the invasion of leukocytes into the inflamed mucosa. Thus we conclude that functional classic IL-6R signalling in IECs is not involved in AOM-DSS-driven carcinogenesis. En masse we have shown that the IL-6 classic signalling pathway does not activate epithelial pSTAT3 and is not essentially involved in the orchestration of STAT3-driven regenerative responses in the context of intestinal inflammation. This finding contrasts observations made in epithelial cancer cells, where IL-6 signalling induces pSTAT3 activation and contributes to epithelial cancer proliferation.^41^ Indeed, we observed in our tested colon cancer cell line a great variety of IL-6 classic signalling responsiveness (data not shown). This finding leaves room for the speculation that classic IL-6 signalling is not an essential epithelial growth signal, but is rather acquired as a second proliferative signal during malignancy. With regard to regenerative response in acute or chronic inflammatory disorder we have shown that interception of the epithelial classic IL-6 signalling does not disrupt epithelial growth and mucosal healing.

Therefore our data do not support the clinical preoccupation about intestinal perforations as a complication in the therapeutic IL-6 interference, but rather come to the conclusion that the IL-6 classic signalling is indispensable for intestinal epithelial regeneration.
